# Effects of Trace Mineral Injections on Measures of Growth and Trace Mineral Status of Primiparous Cows and their Calves

**DOI:** 10.1093/tas/txae068

**Published:** 2024-05-15

**Authors:** Gracia P Hernandez, Matheus F L Ferreira, Aline C R Santos, David Bohnert, Juliana Ranches

**Affiliations:** Eastern Oregon Agricultural Research Center, Oregon State University, Burns, OR 97720, USA; Louisiana State University, Hill Farm Research Station, Homer, LA 71040, USA; Eastern Oregon Agricultural Research Center, Oregon State University, Burns, OR 97720, USA; Eastern Oregon Agricultural Research Center, Oregon State University, Burns, OR 97720, USA; Eastern Oregon Agricultural Research Center, Oregon State University, Burns, OR 97720, USA

**Keywords:** ceruloplasmin, cortisol, haptoglobin, trace minerals, preconditioning, rangelands

## Abstract

The objective of this study was to evaluate the effects of injectable trace minerals (ITM) administrations at strategic moments in the beef cattle production cycle. At calving, 50 primiparous cows (Angus × Hereford) and their calves were randomly assigned to 1 of 2 treatments: 1) ITM: cattle assigned to the ITM treatment received an ITM injection at calving and a subsequent administration at breeding (cattle over 2 yr: 1.0 mL/90 kg body weight [BW]; calves: 1.0 mL/45 kg BW); or 2) Control: cattle assigned to the control treatment were administered with saline following the same procedure as the cattle assigned to the ITM treatment. Body weight, blood, and liver samples were collected from dams and calves at multiple time points to evaluate the growth and mineral status of cow–calf pairs. All variables were analyzed using the MIXED procedure of SAS. A treatment effect (*P =* 0.02) was observed for Cu liver concentration of primiparous cows at breeding. Cows assigned to ITM treatment had greater Cu status than cohorts assigned to Control treatment. No treatment effects were observed for the mineral status or growth of calves. The administration of ITM to primiparous cows enhanced Cu status when grazing Cu forages scarce of Cu.

## Teaser

Injectable trace minerals (ITM) administered to primiparous cows and their calves at strategic moments during the beef production cycle resulted in enhanced mineral status of the dams. Females administered with ITM had greater Cu liver concentration when compared to dams assigned to control treatment.

## Introduction

Trace minerals are known to be important for physiological functions related to growth, reproduction, and immunity in livestock (Suttle, 2010). For grazing beef cattle, forage is the primary source of trace minerals. However, in some regions forage trace mineral concentrations are not sufficient to satisfy the trace mineral requirements of cattle, requiring a supplementation program, such as free-choice loose mineral mixes, trace-mineral-fortified salt blocks, or trace-mineral-fortified energy and protein supplements (Arthington and Ranches, 2021). Although those traditional methods of supplying trace minerals to cattle are efficient, they are not always suitable for all production environments such as the extensive rangelands found in the western of United States.

In eastern Oregon, from spring to fall, cattle graze native forages (*Stipa* spp., *Pseudoroegneria spicata, Poa secunda, Artemisia tridentata* subsp. *wyomingensis*) on U.S. Forest Service and/or Bureau of Land Management grazing allotments. The trace mineral concentration of forages on these allotments is often below the requirements of mature and growing cattle, especially for Cu, Se, and Zn (Carter et al., 1970; Ganskopp and Bohnert, 2003). Additionally, the stocking capacity of these pastures is often limited due to highly variable annual forage production (often ranging from 200 to 1,200 kg/ha; Sneva, 1982) which results in low numbers of cattle per acre (Turner and DelCurto, 1991; Ganskopp and Bohnert, 2001) when compared to more productive environments. Due to logistical constraints associated with these extensive rangeland pastures, including accessibility, traditional trace mineral supplementation, such as free-choice supplements, is limited or not provided at all (Arthington and Ranches, 2021).

The use of injectable trace minerals (ITM) can be a tool when traditional mineral supplementation strategies are limited. Another key advantage of ITM is the ability to conveniently plan the delivery of a known amount of trace minerals over specific periods, avoiding problems with inconsistent intake of traditional mineral supplements and consequently providing the ability to boost the trace mineral status of these animals during challenging events, such as calving, branding, breeding, weaning, and transportation (Arthington et al., 2014).

In beef cattle operations, breeding, calving, and weaning are the most important, and perhaps most critical periods of the entire production cycle, especially for the still developing heifer (Cushman et al., 2013; Day and Nogueira, 2013). Heifer development is a crucial strategy for the continuity of the herd and consequently the success of operation (Moorey and Biase, 2020), and this topic has been widely studied in the past, however, most of the research focused on cattle growth (Moriel et al., 2012, 2020) and on the physiology of puberty achievement (Patterson et al., 1992; Funston et al., 2012; Day and Nogueira, 2013), with limited research post first parturition. Furthermore, there is paucity of literature that has evaluated the mineral status of growing females (pre- and/or post-first parturition), which can be highly influenced by the greater trace mineral transfer from dams to fetus happening in the last trimester of gestation (Hidiroglou, 1980; Hidiroglou and Knipfel, 1981). Because of the particularities of this animal category, the administration of ITM to primiparous females and their calves at specific periods may be warranted to improve mineral status and, consequently, production outcomes of dams and their offspring.

Therefore, the objective of this study was to evaluate the effects of ITM on growth and mineral status of primiparous cows and their calves, when ITM was provided to both at calving and subsequently at breeding, both critical periods in cattle production systems. Thus, we hypothesized that primiparous cows and their calves receiving ITM at calving and at breeding will have enhanced mineral status and growth when compared to dams and calves not administered with ITM.

## Material and Methods

All animal care and handling procedures were approved by the Institutional Animal Care and Use Committee of Oregon State University (IACUC-2021-0144). The study was conducted at the Eastern Oregon Agricultural Research Center—Oregon State University (EOARC, Burns, OR; 43°51ʹ86″N to 119°02ʹ15″W).

### Animal Management and Treatment Allocation

During the calving season, 50 primiparous cows (Angus × Hereford) were selected and randomly assigned at calving (day -200) with their calves to 1 of 2 treatments: 1) ITM: primiparous cows assigned to the ITM treatment received an ITM injection at calving and subsequent ITM injection at breeding (1 mL/90 kg BW; Multimin 90; Fort Collins, Colorado). Similarly, calves born to these dams received an ITM injection at birth and at breeding (calves: 1.0 mL/45 kg BW); 2) Control: primiparous cows assigned to the control treatment followed the same procedure as the cattle assigned to the ITM treatment, however, these primiparous cows and calves were injected with saline solution at calving/birth and at breeding. Saline injections were administered to cows assigned to control treatment to ensure that all animals were treated similarly, and to avoid in any possible site reaction differences between treatments (Caramalac et al., 2021). The ITM utilized contained 60, 10, and 15 mg/mL of Zn, Mn, and Cu, as disodium EDTA chelates, and 5 mg/mL of Se, as sodium selenite. The ITM administration followed the manufacture recommendation, however, the timing of application was chosen to be concomitant with other management activities, in order to be a convenient timing of application for producers. According to the manufacture, this ITM is recommended to be administrated to females every 3 mo, and especially 4 wk prior to calving, while calves should be administered at birth and at 3 mo old or/and at weaning. However, by study design, ITM administration was performed at calving and at breeding, which was planned to coincide to when producers would be handling cattle, resulting in ITM administration being approximately 110 d apart.

During calving season, primiparous cows were maintained in a dry-lot and were monitored every 2 h for parturition signals as routinely conducted at EOARC. Primiparous cows were fed alfalfa hay (*Medigaco sativa;* 14.5% crude protein (CP), 48% neutral detergent fiber (NDF), 67% total digestible nutrients (TDN), 5 ppm, 59 ppm, 0.11 ppm, and 18 ppm, respectively, for Cu, Mn, Se, and Zn) ad libitum once a day. All primiparous cows enrolled in the study successfully calved during a 40-d calving season, resulting in 24 pairs assigned to ITM treatment and 26 pairs assigned to Control treatment.

At calving (day -200), treatment was administrated within 12 h after birth. Liver samples were collected from a subgroup of primiparous cows and calves (*n* = 12 pairs/treatment) for analysis of mineral concentration, and birth weights were collected from all calves using an electronic scale. After treatment administration and sample collections at birth, cow–calf pairs were move to a pasture (approximately 24 ha; recently harvested for hay) and managed as a single group at EOARC until the turnout (summer grazing). During this period, cow–calf pairs were managed as a single group and were fed meadow hay and supplemented with alfalfa hay. Pairs had free access to water and free-choice loose mineral supplement (EOARC Burns Mineral; 13.4% Ca, 10.28% P, 6.14 % Na, 0.74% K, 2.2% Mg, 0.86% S, 922 ppm Mn, 6,000 Zn, 3,275 ppm Cu, 62 ppm Co, 143 ppm Se).

At turnout (day -130; calf age: 62  ± 11 d), body weights (BW) were collected from primiparous cows and their calves. Additionally, at turnout calves were vaccinated (7-way clostridium, infectious bovine rhinotracheitis (IBR) virus, bovine viral diarrhea (BVD) Type 1 virus, parainfluenza 3 (PI_3_) virus, bovine respiratory syncytial virus (BRSV) and *Histophilus somni*) and branded with a hot iron. After turnout processing, cow–calf pairs were transported to the Northern Great Basin Experimental Range (NGBER; approximately 75 km). At the NGBER pairs were kept as a single group grazing native rangeland pastures (*Stipa* spp., *Pseudoroegneria spicata*, *Poa secunda*, *Artemisia tridentata* subsp. *wyomingensis*) with free access to water and free-choice loose mineral supplement (EOARC Burns Mineral). Mineral supplement was replaced, as needed, however intake was no recorded.

Approximately 40 d after the turnout, primiparous cows were enrolled in a 60-d breeding season, where they were artificially inseminated, followed by bull exposure. At breeding (day -90), a second administration of treatments (identical to the initial administration at calving) was provided to primiparous cows and their calves. The timing of treatment administrations were selected to coincide with those two management practices (calving and breeding), where cattle would be routinely handled. Additionally, at breeding, BW and liver (subgroup *n* = 12 pairs/treatment) samples were collected from cows and calves. After the second treatment administration and sample collections, pairs were returned to pastures and managed as previously described until weaning (day 0).

At weaning (day 0) BW was collected from calves and dams, and blood, and liver (subgroup *n* = 12 calves/treatment) samples were collected from calves. Immediately after weaning calves were transported on a livestock trailer back to the EOARC (approximately 75 km). Upon arrival at EOARC, calves were maintained as a single group for a 45-d preconditioning phase prior to be shipped to the feedlot. Calves were maintained in a recently harvested pasture (*Medicago Sativa*) and had free access to water and a chopped (approximately 10 cm) alfalfa-grass hay (14.5 % CP, 48% NDF, 67% TDN, 5 ppm, 59 ppm, 0.11 ppm, and 18 ppm, respectively, for Cu, Mn, Se, and Zn).

During the preconditioning phase, additional BW was measured and blood samples were collected on days 1, 3, 7, 14, 21, 44 and 45 (BW only) postweaning. Calf BW on days 0 and 1 were averaged and considered calf weaning weight, whereas final BW was obtained by the average of BW collected on days 44 and 45. Full BW was accessed instead of shrunk BW to minimize weight loss, and avoid shrink-induced stress effects on blood parameters evaluated (Lay et al., 1996).

### Sample Collections

Blood samples were collected via jugular vein using commercial heparinized vacuum tubes for plasma harvest and tubes containing no additive for serum harvest (BD Vacutainer, 10 mL; Becton, Dickinson and Company, Franklin Lakes, NJ). Blood samples were placed on ice immediately following collection and centrifuged at 2,500 × *g* for 30 min at 4°C for plasma harvest. Plasma samples were frozen at −20 °C and stored at −80 °C until further analysis.

Liver tissue was collected by a trained technician using techniques previously described by Arthington and Corah (1995). Samples were collected between the 10th and 11th intercostal space using a Tru-Cut biopsy needle (CareFusion, 14-gauge × 15 cm; Becton Dickinson, Vernon Hills, IL, USA). Four core tissue samples were collected from each animal. Following collection, samples were frozen at −20 °C and sent to an analytical laboratory for mineral analyses (Michigan State University, Animal Health Diagnostic Laboratory, Lansing, MI, USA).

### Laboratory Analysis

Plasma cortisol, ceruloplasmin, and haptoglobin were analyzed in this study as markers of stress and inflammatory responses, as previously evaluated by our group (Ranches et al., 2021). These markers when elevated have been reported to negatively impact animal growth and immunity, while improved trace mineral status at weaning have been reported to ameliorate these negative outcomes (Carroll and Forsberg, 2007; Ranches et al., 2021).

Plasma cortisol concentrations were measured using chemiluminescent enzyme immuno-assays (Immulite 1000; Siemens Medical Solutions Diagnostics, Los Angeles, CA) with an intra-assay CV of 8.34%.

Plasma ceruloplasmin oxidase activity was measured in duplicate samples using colorimetric procedures previously described (Demetriou et al., 1974). Ceruloplasmin concentrations are expressed as mg/dL as described by King (1965). The intra- and inter-assay CV were 3.03% and 9.46%, respectively.

Plasma haptoglobin concentrations were determined in duplicate samples by a biochemical assay measuring the haptoglobin–hemoglobin complex by estimating differences in peroxidase activity (Makimura and Suzuki, 1982). Results were obtained as arbitrary units resulting from reading plates at 450 nm (VersaMax Tunable EXT). The same quality control standards used in the biochemical assay were analyzed by quantitative determination of bovine haptoglobin in plasma (bovine haptoglobin ELISA test kit; Life Diagnostics, Inc., West Chester, PA, USA). The concentration of haptoglobin, based on the ELISA assay, ranged from 0.03 (low control) to 0.95 mg/mL (high control) with an intra-assay coefficient of variability (CV) of 1.26%. The ELISA standard curve was used to convert the arbitrary units obtained from the biochemical procedures into mg/mL with the least detectable value of 0.03 mg/mL (Cooke and Arthington, 2013). The intra- and inter-assay CV were 3.84% and 10.59%, respectively.

### Statistical Analysis

For data analysis, primiparous cow and calf was considered the experimental unit in this study. All variables were analyzed using the MIXED procedure of SAS (SAS Inst. Inc., Cary, NC). Briefly, the model statement included treatment, day, and possible interactions, and day was included in the repeated statement. Calf age and sex were tested and removed from model as those had no effect. Dam initial body weight was used as covariate for the analysis of subsequent body weight. Compound symmetry covariance structure was used for the repeated measures analyses, as this covariance structure generated the lowest Akaike information criterion. Data were separated using PDIFF if a significant preliminary F test was detected. Significance was set at *P *≤ 0.05, and tendencies if *P > *0.05 and ≤0.10.

## Results

No effects of treatment (*P =* 0.67) or a treatment × time *(P = *0.97) were observed for cow body weight during the study. Only an effect of time (*P < *0.0001) was observed with a reduction in BW over time regardless of treatment (Table 1).

**Table 1.** Body weight of primiparous cows administrated or not (Control) with injectable trace minerals (ITM)

	Treatments^1^			
Item	Control	ITM	SEM	*P* value
Turnout, kg	416^a^	416^a^	5.9	1.00
Breeding, kg	421^a^	419^a^	5.9	1.00
Weaning, kg	386^b^	384^b^	5.9	0.999

^a,b^means within the same treatment with different superscripts differ over time (P < 0.0001).

^1^During the calving season, 50 primiparous cows and their calves were randomly assigned to 1 of 2 treatments: 1) Injectable trace minerals (ITM): primiparous cows assigned to the ITM treatment received an ITM injection at the calving and breeding (cattle over 2 yr: 1 mL/90 kg BW). Similarly, calves born to these cows received an ITM injection at birth and breeding (1.0 mL/45 kg BW); 2) Control: cattle assigned to the control treatment followed the same procedure as the cattle assigned to the ITM treatment, however, these dams and calves were injected with saline solution.

For liver Se concentration of cows there were no effects of treatment (*P =* 0.59) or treatment × time (*P =* 0.64). There was an effect of time (*P =* 0.03) for liver Se concentration where liver Se concentration tended (*P =* 0.07) to increase over time for cows assigned to the control treatment (Table 2).

**Table 2.** Liver trace mineral concentration of primiparous cows administrated or not (saline) with injectable trace minerals (ITM)

	Treatments^1^			
Item	Saline	ITM	SEM	*P* value
Initial Se, mg/kg^2^	0.69	0.75	0.059	0.48
Breeding Se, mg/kg^3^	0.85^*^	0.86	0.066	0.92
Initial Cu, mg/kg^2^	33.4^b^	50.8^b^	9.27	0.21
Breeding Cu, mg/kg^3^	50.1^a^	85.0^a^	9.96	0.02
Initial Mn, mg/kg^2^	9.57^a^	10.3^a^	0.612	0.44
Breeding Mn, mg/kg^3^	6.71^b^	6.93^b^	0.657	0.81
Initial Zn, mg/kg^2^	273^a^	286^a^	12.9	0.50
Breeding Zn, mg/kg^3^	214^b^	207^b^	13.9	0.73

^a,b^means within the same treatment with different superscripts differ over time.

^*^There was an effect of time (*P = *0.03) for liver Se concentration where liver Se concentration tended (*P = *0.07) to increase for cows assigned to the Saline treatment.

^1^During the calving season of 2021, 50 primiparous cows and their calves were randomly assigned to 1 of 2 treatments: 1) Injectable trace minerals (ITM): primiparous cows assigned to the ITM treatment received an ITM injection at the calving and breeding (cattle over 2 yr: 1 mL/90 kg BW). Similarly, calves born to these cows received an ITM injection at birth and breeding (1.0 mL/45 kg BW); 2) Saline: cattle assigned to the saline treatment followed the same procedure as the cattle assigned to the ITM treatment, however, these dams and calves were injected with saline.

^2^Initial liver samples were collected at birth to evaluate mineral status of cows. Adequate mineral status is reported by the Michigan State University Veterinary Diagnostic Laboratory as Cu: 40 to 650 mg/kg; Se: 0.60 to 3.30 mg/kg; Mn: 5.50 to 15.00 mg/kg; Zn: 90 to 500 mg/kg.

^3^A second liver sample was collected at breeding to evaluate mineral status of cows.

There was an effect of treatment (*P =* 0.02) for Cu liver concentration at breeding where cows assigned to ITM treatment had greater liver Cu concentration than cows assigned to Control. There was a time effect (*P < *0.0001) where Cu liver concentration increased over time regardless of treatment, but there was no effect of treatment × time (*P =* 0.11; Table 2).

For liver Mn concentration of cows there were no effects of treatment (*P =* 0.55) or treatment × time (*P =* 0.61). There was an effect of time (P < 0.0001) for liver Mn concentration, where concentration decreased over time regardless of treatment (Table 2).

For liver Zn concentration of cows there were no effects of treatment (*P = *0.82) or treatment × time (*P =* 0.46). There was an effect of time (P < 0.0001) for liver Zn concentration, where concentration decreased over time regardless of treatment (Table 2).

No effects of treatment (*P = *0.41) or a treatment × time (*P = *0.80) were observed for calf BW during the study. Only an effect of time (*P < *0.0001) was observed where calf BW increased over time regardless of treatment (Table 3). However, calves assigned to ITM treatment consistently had a numerical advantage on BW when compared to calves assigned to Control. At the end of the preconditioning phase, calves assigned to ITM treatment were 4 kg heavier than calves assigned to Control treatment.

**Table 3.** Body weight of calves administrated or not (saline) with injectable trace minerals (ITM)

	Treatments^1^			
Item	Control	ITM	SEM	*P* value
Birth, kg	31^e^	30^e^	4.6	1.00
Turnout, kg	77^d^	79^d^	4.6	0.99
Breeding, kg	114^c^	117^c^	4.7	0.99
Weaning, kg	176^b^	179^b^	4.7	0.99
End Preconditioning, kg	215^a^	219^a^	4.7	0.98

^a,b,c,d,e^means within the same treatment with different superscript differ overtime *(P < 0.0001).*

^1^During the calving season of 2021, 50 primiparous cows and their calves were randomly assigned to 1 of 2 treatments: 1) Injectable trace minerals (ITM): cows assigned to the ITM treatment received an ITM injection at the calving and breeding (cattle over 2 yr: 1 mL/90 kg BW). Similarly, calves born to these cows received an ITM injection at birth and breeding (1.0 mL/45 kg BW); 2) Control: cattle assigned to the control treatment followed the same procedure as the cattle assigned to the ITM treatment, however, these dams and calves were injected with saline solution.

For liver Se concentration of calves there were no effects of treatment (*P =* 0.89) or treatment × time (*P =* 0.85). There was an effect of time (*P* < 0.0001) for liver Se concentration where liver Se decreased from birth to breeding and increased from breeding to weaning (Table 4).

**Table 4.** Liver trace mineral concentration of calves administrated or not (saline) with injectable trace minerals (ITM)

	Treatments^1^			
Item	Saline	ITM	SEM	*P* value
Initial Se (mg/kg)^2^	1.69^a^	1.84^a^	0.287	0.60
Breeding Se (mg/kg) ^3^	0.69^b^	0.68^b^	0.317	0.97
Weaning Se (mg/kg)^3^	1.69^a^	1.62^a^	0.293	0.81
Initial Cu (mg/kg)^2^	251^a^	191^a^	32.1	0.07
Breeding Cu (mg/kg)^3^	52.7^b^	50.2^b^	35.5	0.94
Weaning Cu (mg/kg)^3^	188^a^	175^a^	32.6	0.94
Initial Mn (mg/kg)^2^	3.95^b^	4.25^b^	0.795	0.70
Breeding Mn (mg/kg)^3^	5.36^b^	5.51^b^	0.878	0.86
Weaning Mn (mg/kg)^3^	7.32^a^	6.53^a^	0.759	0.32
Initial Zn (mg/kg)^2^	584^a^	637^a^	45.6	0.24
Breeding Zn (mg/kg)^3^	238^b^	232^b^	50.7	0.90
Weaning Zn (mg/kg)^3^	240^b^	265^b^	45.6	0.60

^a,b^means within the same treatment with different superscript differ overtime.

^1^During the calving season of 2021, 50 primiparous cows and their calves were randomly assigned to 1 of 2 treatments: 1) Injectable trace mineral (ITM): primiparous cows assigned to the ITM treatment received an ITM injection at the calving and breeding (cattle over 2 yr: 1 mL/90 kg BW). Similarly, calves born to these cows received an ITM injection at birth and breeding (1.0 mL/45 kg BW); 2) Control: cattle assigned to the control treatment followed the same procedure as the cattle assigned to the ITM treatment, however, these dams and calves were injected with saline solution.

^2^Initial liver samples were collected at birth to evaluate mineral status of calves. Adequate mineral status is reported by the Michigan State University Veterinary Diagnostic Laboratory as Cu: 40 to 650 mg/kg; Se: 0.60 to 3.30 mg/kg; Mn: 5.50 to 15.00 mg/kg; Zn: 90 to 500 mg/kg.

^3^A second liver sample was collected at breeding and third liver sample was collected at weaning to evaluate mineral status of calves.

For liver Cu concentration of calves there were no effects of treatment (*P =* 0.12) or treatment × time (*P =* 0.48). There was an effect of time (*P < *0.0001) for liver Cu concentration where liver Cu decreased from birth to breeding and increased from breeding to weaning (Table 4).

For liver Mn concentration of calves there were no effects of treatment (*P =* 0.81) or treatment × time (*P =* 0.58). There was an effect of time (P < 0.0001) for liver Mn concentration where liver Mn was maintained from birth to breeding and increased from breeding to weaning (Table 4).

For liver Zn concentration of calves, there were no effects of treatment (*P =* 0.38) or treatment × time (*P = *0.68). There was an effect of time (*P < *0.0001) for liver Zn concentration where liver Zn decreased from birth to breeding and remained the same until weaning (Table 4).

An effect of day (*P < *0.0001) was observed for plasma cortisol concentration at and postweaning, but no effects of treatment (*P = *0.30) or the possible interactions (*P =* 0.81) were observed (Table 5). Plasma cortisol concentration peaked (*P ≤ *0.04) at weaning as expected, and increased again on day 14.

**Table 5.** Blood markers of calves at weaning and during preconditioning

	Treatments^1^			*P* value		
Item	Control	ITM	SEM	Treatment	Day	Trt × Day
Cortisol, ng/Ml^2^	1.18	1.28	0.07	0.30	<0.0001	0.81
Haptoglobin, µg/mL^3^	0.84	0.83	0.05	0.89	<0.0001	0.72
Ceruloplasmin, mg/mL^4^	30.7	29.9	0.74	0.41	<0.0001	0.23

^1^During the calving season of 2021, 50 primiparous cows and their calves were randomly assigned to 1 of 2 treatments: 1) Injectable trace mineral (ITM): primiparous cows assigned to the ITM treatment received an ITM injection at the calving and breeding (cattle over 2 yr: 1 mL/90 kg BW). Similarly, calves born to these cows received an ITM injection at birth and breeding (1.0 mL/45 kg BW); 2) Control: cattle assigned to the control treatment followed the same procedure as the cattle assigned to the ITM treatment, however, these dams and calves were injected with saline solution.

^2^Plasma cortisol concentrations were measured using chemiluminescent enzyme immuno-assays (Immulite 1000; Siemens Medical Solutions Diagnostics, Los Angeles, CA).

^3^Plasma haptoglobin concentrations were determined in duplicate samples by a biochemical assay measuring haptoglobin–hemoglobin complexing by the estimation of differences in peroxidase activity (Makimura and Suzuki, 1982).

^4^Plasma ceruloplasmin oxidase activity was measured in duplicate samples using colorimetric assay (Demetriou et al., 1974).

An effect of day (*P < *0.0001) was observed for plasma ceruloplasmin concentration, but no effects of treatment (*P = *0.41) or the possible interactions (*P =* 0.23) were observed (Table 5). Plasma ceruloplasmin concentrations were greatest on days 3 and 7 postweaning.

An effect of day (*P < *0.0001) was observed for plasma haptoglobin concentration, but no effects of treatment (*P = *0.89) or the possible interaction (*P =* 0.72) were observed (Table 5). Plasma haptoglobin concentration increased on day 1 after weaning and peaked on day 3 postweaning followed by a decrease in plasma haptoglobin concentration.

## Discussion

The use of ITM is a tool to enhance and boost mineral status of cattle when traditional mineral supplementation strategies are challenging, or when a fast increase in mineral status is required. With that rational, the use of ITM is often utilized prior to stressful management practices such as branding, breeding, weaning, and transportation (Arthington and Ranches, 2021; Palomares, 2022).

In the current study, no treatment effects were observed for dam BW, which is similar to what has been observed for other cattle categories when using ITM (Mundell et al., 2012; Arthington et al., 2014; Vedovatto et al., 2019). There is a paucity of literature that have adopted the same ITM administration management as the one adopted in this study (calving and breeding). However, Arthington et al. (2014) working with developing heifers, which most closely resemble the cattle category used in this study, reported improved average daily gain when heifers were administered with ITM prior to puberty achievement. The lack of treatment effect on cow BW in the current study is likely associated to the adequate nutritional status observed for these cows throughout the study.

Previous research have demonstrated that the transfers of trace mineral from dam to fetus via placenta happens over the duration of gestation but tends to intensify during late gestation (Hidiroglou, 1980; Hidiroglou and Knipfel, 1981). This transfer can reduce the dams stores of trace minerals, therefore the use of ITM administration at calving is a reasonable management practice as improvements in trace mineral status have been constantly reported for cattle of all categories when administrated with ITM (Arthington and Havenga, 2012; Arthington et al., 2014; Palomares et al., 2016).

Except for Cu, cows had adequate trace mineral status at the beginning of the study, which was maintained throughout the study regardless of treatment (40.0 to 650.0 mg/kg, 0.60 to 3.30 mg/kg, 5.50 to 15.00, 90.0 to 500.0 mg/kg, respectively, for Cu, Se, Mn, and Zn; VDL MSU reference ranges; Herdt and Hoff, 2011). At breeding cows had adequate Cu status, however, cows assigned to ITM treatment had greater liver Cu concentration at breeding than cows assigned to Control treatment. During this phase, cows were grazing native rangelands, which have been reported to have notoriously low Cu concentrations (1.75 mg/kg ± 0.11; Ganskopp and Bohnert, 2001, 2003) and not compatible with cattle requirements (NASEM, 2016). Although no trace minerals deficiencies were observed, the ITM administration scheme adopted in this study aided cows to maintain an adequate mineral status (liver Cu concentration > 40.0 mg/kg) until the breeding season. Although out of the scope of this study, improved mineral status at breeding have been associated with improved reproductive performance (Vedovatto et al., 2019), making the use to ITM a compelling tool for locations where trace mineral concentration of forages does not meet cattle requirements.

Surprisingly no improvement in mineral status with ITM administration was observed for calves in the current study. Using somewhat similar ITM administration, Arthington et al. (2014) reported improved Cu and Se status of calves assigned to ITM treatment when calves were injected with ITM or saline within 24h after birth, and again when calves were 100 and 200 d old. The timing of treatment administration and the sampling interval of the current study might have hindered our ability to demonstrate any improvement in trace mineral status of calves assigned to ITM as observed by others (Arthington and Havenga, 2012; Arthington et al., 2014; Palomares et al., 2016). However, regardless of treatment assignment calves maintained adequate mineral status throughout the study (40.0 to 650.0, 0.60 to 3.30, 5.50 to 15.00, 90.0 to 500.0 mg/kg, respectively, for Cu, Se, Mn, and Zn; VDL MSU reference ranges; Herdt and Hoff, 2011). Additionally, although calves were exposed to stress such as branding, transportation and weaning, calves in this study had minor management and environmental stressor, which could have reduced the need to mobilize trace mineral reserves.

Further, the administration of ITM to calves at birth and breeding did not impact any of the blood markers evaluated in this study. As mentioned previously, calves were under minor management and environmental stressors therefore may not requiring to develop a strong response to these stressors. Others have reported similar response where ceruloplasmin and haptoglobin concentrations were unaffected by ITM administration (Genther and Hansen, 2014; Caramalac et al., 2021), while Arthington et al. (2014) reported increased concentration of ceruloplasmin and haptoglobin concentrations for heifers administered ITM prior to weaning and transport, however the authors associated this increase in the acute-phase proteins, especially ceruloplasmin, to the greater trace mineral status of those calves, which was not observed in the current study.

## Conclusion

In summary, the use of ITM to primaparous cows and their offspring at calving and at breeding resulted improved Cu status of cows at breeding. The use of ITM administration concomitant to cattle handling events (calving and breeding) might be a strategy to enhance trace mineral status of cattle grazing forages that do meet cattle requirements, as observed by the increased Cu status of cows in this study.
